# Mapping the tuberculosis scientific landscape among BRICS countries: a bibliometric and network analysis

**DOI:** 10.1590/0074-02760190342

**Published:** 2020-03-16

**Authors:** Kamaiaji Castor, Fabio Batista Mota, Roseli Monteiro da Silva, Bernardo Pereira Cabral, Ethel Leonor Maciel, Isabela Neves de Almeida, Denise Arakaki-Sanchez, Kleydson Bonfim Andrade, Vadim Testov, Irina Vasilyeva, Yanlin Zhao, Hui Zhang, Manjula Singh, Raghuram Rao, Srikanth Tripathy, Glenda Gray, Nesri Padayatchi, Niresh Bhagwandin, Soumya Swaminathan, Tereza Kasaeva, Afrânio Kritski

**Affiliations:** 1 Fundação Oswaldo Cruz-Fiocruz, Centro de Estudos Estratégicos, Rio de Janeiro, RJ, Brasil; 2 Universidade Federal do Espírito Santo, Laboratório de Epidemiologia, Vitória, ES, Brasil; 3 Universidade Federal de Minas Gerais, Faculdade de Medicina, Laboratório de Pesquisa em Micobactérias, Belo Horizonte, MG, Brasil; 4 Ministério da Saúde, Programa Nacional de Controle da Tuberculose, Brasília, DF, Brasil; 5 National Medical Research Centre of Pthtisiopulmonology and Infection Diseases, MoH, Moscow, Russian Federation; 6 National Centre for Tuberculosis Control and Prevention, China CDC, Changping District, Beijing, China; 7 Indian Council of Medical Research, New Delhi, India; 8 Government of India, Ministry of Health and Family Welfare Central TB Division, New Delhi, India; 9 National Institute for Research in Tuberculosis, Chennai, India; 10 University of KwaZulu-Natal, Doris Duke Medical Research Institute, South African Medical Research Council HIV-TB Pathogenesis and Treatment Research Unit, Congella, South Africa; 11 Phthisiology, Central TB Research Institute, Moscow, Russian Federation; 12 Universidade Federal do Rio de Janeiro, Faculdade de Medicina, Programa Acadêmico de Tuberculose, Rio de Janeiro, RJ, Brasil; 13 World Health Organization, Geneva, Switzerland

**Keywords:** tuberculosis, scientific landscape, BRICS, bibliometrics, research network analysis

## Abstract

**BACKGROUND:**

The five BRICS (Brazil, Russian, Indian, China, and South Africa) countries bear 49% of the world’s tuberculosis (TB) burden and they are committed to ending tuberculosis.

**OBJECTIVES:**

The aim of this paper is to map the scientific landscape related to TB research in BRICS countries.

**METHODS:**

Were combined bibliometrics and social network analysis techniques to map the scientific publications related to TB produced by the BRICS. Was made a descriptive statistical data covering the full period of analysis (1993-2016) and the research networks were made for 2007-2016 (8,366 records). The bubble charts were generated by VantagePoint and the networks by the Gephi 0.9.1 software (Gephi Consortium 2010) from co-occurrence matrices produced in VantagePoint. The Fruchterman-Reingold algorithm provided the networks’ layout.

**FINDINGS:**

During the period 1993-2016, there were 38,315 peer-reviewed, among them, there were 11,018 (28.7%) articles related by one or more authors in a BRICS: India 38.7%; China 23.8%; South Africa 21.1%; Brazil 13.0%; and Russia 4.5% (The total was greater than 100% because our criterion was all papers with at least one author in a BRICS). Among the BRICS, there was greater interaction between India and South Africa and organisations in India and China had the highest productivity; however, South African organisations had more interaction with countries outside the BRICS. Publications by and about BRICS generally covered all research areas, especially those in India and China covered all research areas, although Brazil and South Africa prioritised infectious diseases, microbiology, and the respiratory system.

**MAIN CONCLUSIONS:**

An overview of BRICS scientific publications and interactions highlighted the necessity to develop a BRICS TB research plan to increase efforts and funding to ensure that basic science research successfully translates into products and policies to help end the TB epidemic.

In 2014, the World Health Assembly endorsed the End TB Strategy with the aim of attaining a 90% reduction in tuberculosis (TB)-related mortality and 80% reduction in disease incidence by 2030, in line with target 3.3 of the Sustainable Development Goals on combating communicable diseases, including TB.^1^ ^,^ ^2^ To achieve these ambitious goals, “research and innovation” was identified as one of the three essential pillars to end the TB epidemic through the discovery of and equitable access to innovative tools and approaches, at both national and global levels.

In 2015, World Health Organization (WHO) released a *Global Action Framework for TB Research* to stimulate, enhance, and facilitate multi-stakeholder actions for a more efficient and impact-oriented TB Research & Development (R&D).^3^ Despite the strong advocacy and call for enhanced TB R&D at both the national and local level, most TB programs have focused on programmatic actions and still face difficulties in incorporating R&D into their work plans. R&D funding agencies are often unlinked from TB programs, requiring collaborative efforts among sectors, thus keeping funding below what is needed to make progress.

The current global research scenario shows that high-income countries focus their priorities on demands or short-term outcomes of a transactional nature. Therefore, the countries most affected by TB (low- and middle-income countries) need to act, show leadership, and invest in TB control as well as research.^4^

Taking into account these scenarios and given the benefits gained per dollar spent on those actions, the need for enhanced TB research has received additional recognition at the highest political levels, as demonstrated by the 2018 political declaration of the United Nations General Assembly High-Level Meeting (UNGA-HLM) on the fight against TB.^5^

The five BRICS countries (Brazil, Russian, Indian, China, and South Africa) bear 49% of the world’s burden of TB, 40% of all TB-related mortality, and more than 60% of the multidrug-resistant TB burden.^6^ ^,^ ^7^ In response, following the BRICS Leaders Xiamen Declaration (2017) addressing the need to improve surveillance of TB and to set up a TB research network (BRICS TB Research Network 2018;^8^ Xiaodong 2017), the BRICS National Tuberculosis Programme (NTP) managers and leaders in academia organised the first BRICS TB Research Network meeting in September 2017 in Rio de Janeiro, Brazil, at which the BRICS countries committed to combat TB and develop a TB research agenda.^9^

While the United States has remained the top producer of TB research for the past two decades, India and China have emerged as the second- and third-leading producers of TB research in recent years. Bibliometric analyses have shown that the average number of TB publications from the BRICS countries has doubled every year since 2007.^10^

This paper describes the evolution of TB-related scientific publications in English produced by BRICS countries between 1993 and 2016. We used bibliometrics and social network analysis to map the scientific publications, the main organisations, the strength of collaboration between these countries, and the distribution of the articles’ research areas.

Although Nafade et al.^10^ also performed a bibliometric analysis for TB, our study differed in methods and coverage. Specifically, we considered only BRICS countries to identify the main organisations and the strength of collaboration among those countries over a longer period (1993-2016) using social network techniques. Nafade et al.^10^ emphasised the importance of collaboration among BRICS countries to increase TB research productivity; our study takes a step in this direction.

## MATERIALS AND METHODS

We combined bibliometrics and social network analysis techniques to map the scientific publications related to TB produced by the BRICS countries. Data from scientific publications (articles only) were collected from Thomson Reuters’ Web of Science Core Collection (WoS). We chose WoS because the copious amount of secondary information available for its indexed papers offered many possibilities for bibliometric analysis. The search was carried out in May 2017 using the advanced search mode of WoS and the following query: (ti = (Tuberculosis or Tuberculoses or “Koch* Disease” or Antitubercular* or Tuberculostatic* or Tuberculoma* or Tuberculous) and cu = (Brazil or Brasil or Russia or India or China or “South Africa”) AND DOCUMENT TYPES: (Article)Indexes=SCI-EXPANDED Timespan=1993-2016.

We selected TB descriptors from the medical subject headings (MeSH) of the National Center for Biotechnology Information (NCBI). To avoid articles whose focus was not TB, we conducted the search using the title (ti) field rather than the topic (ts) field. The ts field encompasses articles’ titles, abstracts, and keywords (from authors and keywords plus) and abstracts and keywords (from authors and keywords plus). “Keywords plus” is based on WoS editorial readings of the titles of the articles’ bibliographic references, which can create a source of possible “garbage” in the analysis.

We restricted our query to only “document type” articles because these are usually more complete and relevant to advanced stages of research.^11^ We selected the “citation indexes” Science Citation Index Expanded index (SCI - EXPANDED) to narrow the focus on research related to biomedical science to avoid publications indexed in social sciences, arts, and humanities (Science Citation Index Expanded). Our period of analysis began in 1993 when WHO declared TB to be a global threat.^12^

Search collected 11,246 records of articles. We imported the raw data from WoS (plain text format) into the VantagePoint v10.0 data/text mining software (Search Technology, Inc. 2017). VantagePoint enabled us to remove duplicates using the ISI unique article identifier tool and remove “Research Areas” not related to the aims of this work (see Appendix); WoS uses “Research Area” to classify articles by subject (Web of Science Core Collection Research Area). This filtering reduced the number of articles to 11,018. We then used VantagePoint to “clean” and normalise the fields “Author Affiliations (Organisation and City and Country)” and “Keywords (authors)” using the List Cleanup tool (general fuzzy logic) associated with manual cleaning. Cleaning and normalisation of such fields are necessary to remove errors and redundancies. This complex procedure produces results that approximate item-by-item manual checking.

Descriptive statistical data covering the full period of analysis (1993-2016) and the research networks were made for 2007-2016 (8,366 records); for the networks, the date restrictions were added to obtain a more reasonable approximation of recent knowledge flow, because a longer sample period might have biased the results by including too much past information. Our aim was to get a picture of contemporary trends in the dissemination of knowledge. This kind of bias occurs when, for example, previously important organisations published extensive research decades ago but no longer play such an important role in the network.^13^

We used the degree-centrality measure to estimate the importance of a given node in a network.^14^ ^,^ ^15^ This metric represents the number of ties to a node. Each tie has a weight defined by the number of co-occurrences between the two nodes it connects. In the networks, the size of each node is a unique and increasing function of the degree - hence the term degree-centrality. The thickness of each edge between two nodes is a unique and increasing function of the weight. The weight is the number of collaborations between two nodes. In summary:

$${Degree}_{i}=\sum_{j\;\neq \;i\;}a_{i,j}\;\;\;(1)$$

$${Thickness}_{i,\;\;j}=Nº\;of\;Collaborations\;between\;i\;and\;j\;\;\;(2)$$

The weight is 1 if there is a connection between the nodes i and j and 0 otherwise. There is said to be a connection between two nodes if they share at least one co-occurrence (in our study, if they shared at least one article). This can be explained by considering country networks. For example, India published at least one article related to 115 other countries, so India had a degree of 115. However, India published 13 articles related to China and 102 related to South Africa. The edges connecting India and South Africa had a greater thickness than those connecting India and China. The number of connections (ties) that a node has plays indicates who has access to different sources of information - those that are connected to several nodes.^14^ ^,^ ^15^ ^,^ ^16^

For the network of research areas, we also assumed the existence of communities, because two or more research areas can be complementary. Given this hypothesis, we chose to explore the modularity properties of the network using the Louvain algorithm^17^ in which nodes that belong to the same community share at least one feature. This does not necessarily mean that two nodes of the same group are strongly connected; after all, the characteristic in common between the nodes of this group could be the absence of connections between themselves and others (in this case, a community with nodes that are dispersed in the network).

The bubble charts were generated by VantagePoint and the networks by the Gephi 0.9.1 software (Gephi Consortium 2010) from co-occurrence matrices produced in VantagePoint. The Fruchterman-Reingold algorithm provided the networks’ layout. The idea was to use a force-directed layout in a way that two nodes in the same cluster would be more likely to be connected by an edge^18^ ^,^ ^19^ ^,^ ^20^ to achieve our objectives.

## RESULTS

**Fig. 1:**
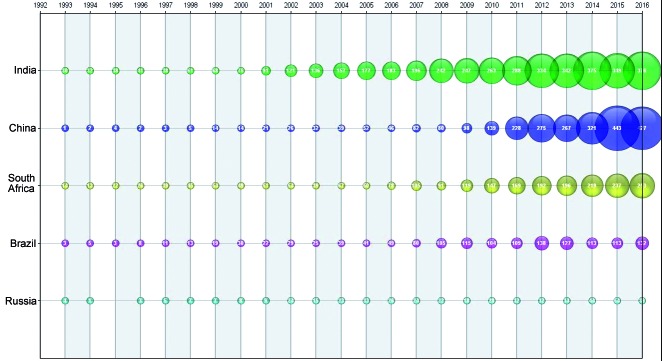
BRICS countries (Brazil, Russian, Indian, China, and South Africa): scientific publications over time (1993-2016).

Between 1993 and 2016, there were 38,315 peer-reviewed, scientific articles covering 150 countries published on health- or medicine-related topics. Among these, there were 11,018 (28.7%) articles related by one or more authors in a BRICS country: India 38.7%; China 23.8%; South Africa 21.1%; Brazil 13.0%; and Russia 4.5% (Fig. 1) (The total was greater than 100% because our criterion for was all papers with at least one author in a BRICS institution).

We measured publications by country using the authors’ institutional affiliations. The number of articles attributed to the BRICS group was less than the individual sum of the articles attributed to the BRICS countries because many articles had co-authors in more than one BRICS country. Similarly, some authors had multiple affiliations and the primary affiliation entered was dependent on how the information was captured. In our study, we only considered the authors’ primary affiliation.

**Fig. 2:**
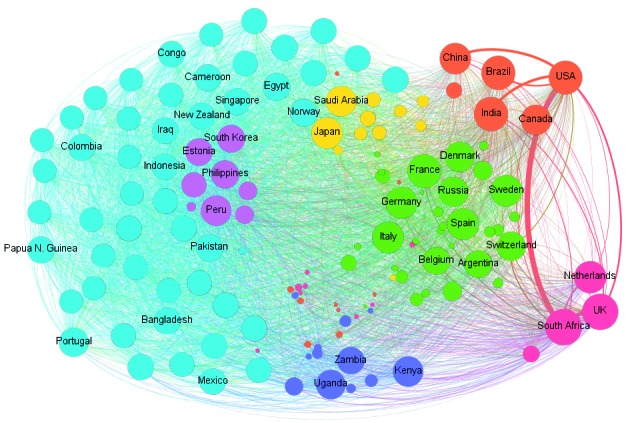
network of BRICS (Brazil, Russian, Indian, China, and South Africa) and extra-BRICS countries (2007-2016). Note: the size of each node is a unique and increasing function of the degree. Degree is the number of ties that a node has.

Fig. 2 shows the network with the nodes reorganised based on the Louvain algorithm, which seeks to obtain a structure of groups/communities in a given network based on modularity measures. In this case, the nodes were grouped on a resolution of 0.6, so seven communities were obtained and represented by distinct colors.^17^ The greater the resolution, the smaller the communities we obtained. We also considered the default resolution (1.0): the overall results did not change for the bigger communities (using the random mode, we found between three and 12 communities). As the results for the main communities were not easily understandable using this choice, we adopted the more suitable resolution of 0.6 to better delineate the main communities.

In Fig. 2, the Orange community can be characterised by a high level of cooperation only with one specific node, USA, and with fewer connections among other BRICS members (China, Brazil, and India), although South Africa has a high cooperation level with European countries (UK and Netherlands) and with the USA. In contrast, the members of the Purple group have a low cooperation level among themselves and with other countries.

**Fig. 3:**
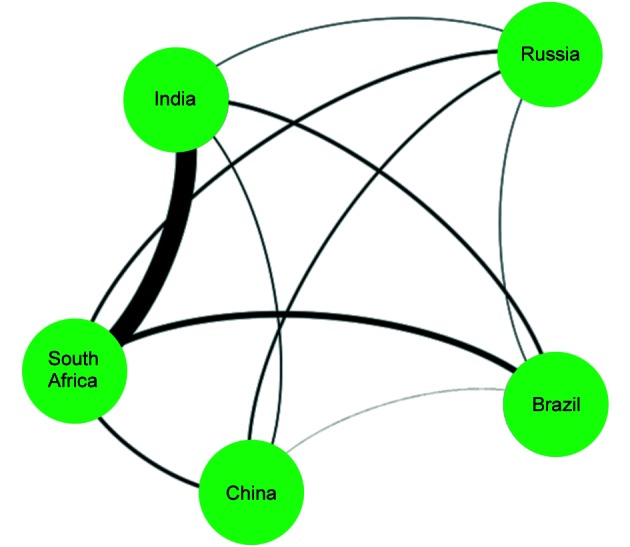
network of scientific production among BRICS countries (Brazil, Russian, Indian, China, and South Africa) (2007-2016).

In Fig. 3, the thickness of the line between India and South Africa shows that these two countries have the highest frequency of collaboration within the BRICS countries.

**Fig. 4:**
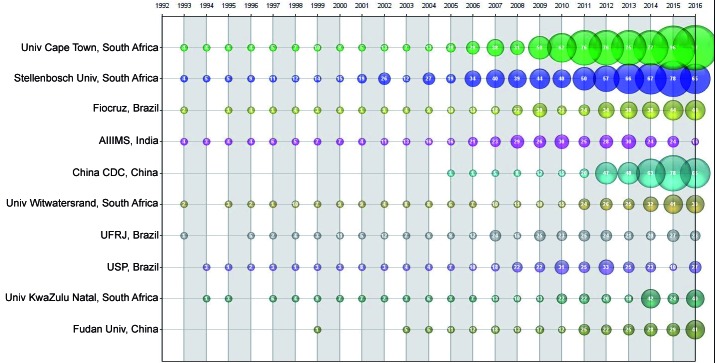
network of BRICS (Brazil, Russian, Indian, China, and South Africa) research organisations: scientific publications (1993-2016)

Among the BRICS countries, we identified the top 10 TB research organisations: University of Cape Town, South Africa (7.4%); Stellenbosch University, South Africa (6.9%); Fiocruz, Brazil (3.5%); All India Institute of Medical Sciences (AIIMS), India (3.4%); China CDC, China (3.3%); University of Witwatersrand, South Africa (2.8%); - Federal University of Rio de Janeiro (UFRJ), Brazil (2.8%); University of São Paulo (USP), Brazil (2.6%); and University of KwaZulu Natal, South Africa (2.6%) (Fig. 4). If other databases such as Scival or Scopus had been used, these results may have been different. The ranking of all TB research organisations and its respective degree centrality is available as Supplementary data.

**Fig. 5:**
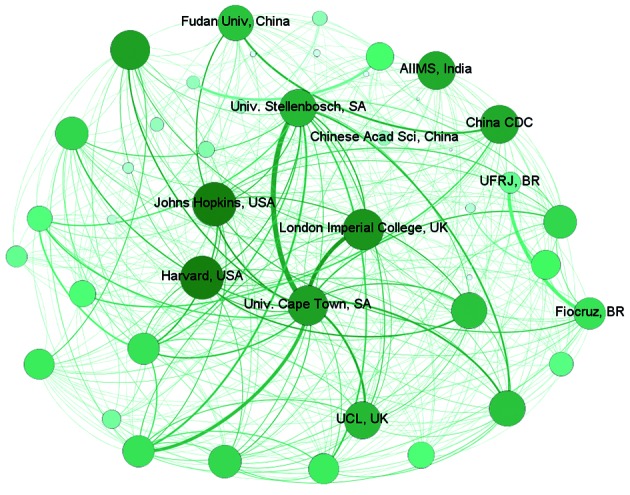
network of tuberculosis (TB) publications within BRICS (Brazil, Russian, Indian, China, and South Africa) research organisations and with other countries’ organisations (2007-2016). Note: the size of each node is a unique and increasing function of the degree. Degree is the number of ties that a node has.

Fig. 5 shows that based on the degree measure, Fudan University from China and AIIMS from India were the top BRICS research organisations. Considering the degree of collaboration, the University of Cape Town had a higher degree and frequency of collaboration, and this collaboration was associated with both UK Universities (e.g., Imperial College, London) and local institutions (e.g., Stellenbosch University). In Brazil, the Oswaldo Cruz Foundation (Fiocruz) and the Federal University of Rio de Janeiro (UFRJ) acted as the national centre of the collaboration network.

**Fig. 6:**
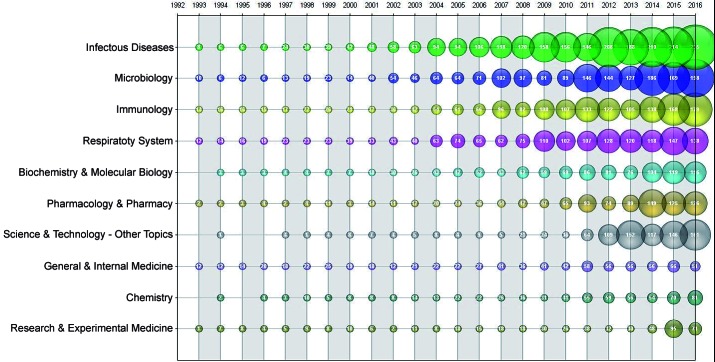
research areas with most publications on tuberculosis (TB) within BRICS (Brazil, Russian, Indian, China, and South Africa) publications (1993-2016).

Our analysis identified the following top research areas: infectious diseases (21.6%); microbiology (16.2%); immunology (15.3%); respiratory system (14.5%); biochemistry and molecular biology (9.6%); pharmacology and pharmacy (9.2%); other science and technology topics (8.1%); general and internal medicine (6.9%); chemistry (5.7%); and research and experimental medicine (4.7%) (Fig. 6).

**Fig. 7:**
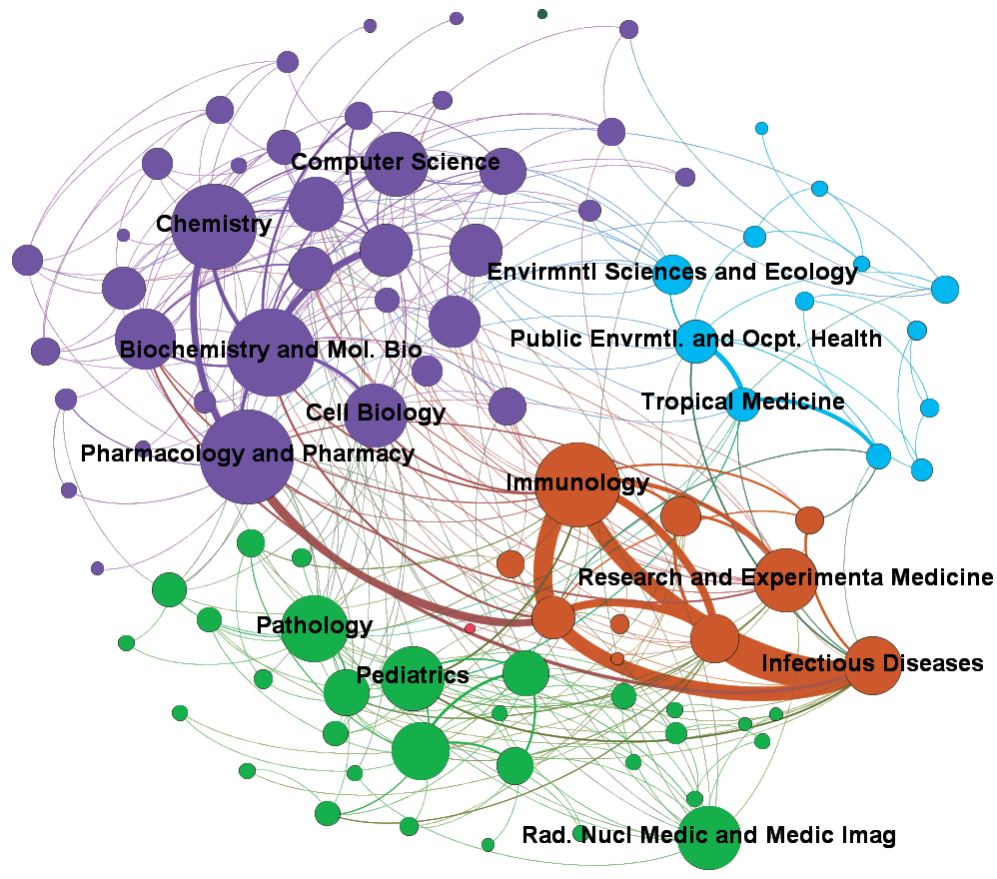
network of research areas (2007-2016). Note: the size of each node is a unique and increasing function of the degree. Degree is the number of ties that a node has. Rad, Nucl Medic and Medic Imag: Radiology Nuclear Medicine and Medical Imaging; Public Envrmtl. and Ocpt. Health: Public Environmental and Occupational Health.

When we evaluated the research areas network (Fig. 7), the nodes were divided by the same Louvain modularity algorithm based on a resolution of 1.0 to group communities and represent them by different colors.^18^ The four main communities identified were these: (a) chemistry, computer science, biochemistry, molecular biology, pharmacology, and cell biology; (b) immunology, research and experimental medicine, and infectious diseases; (c) pathology, pediatrics, and radiological medicine; and (d) environmental and ecological, public environmental, and occupational health and tropical medicine.

**Fig. 8:**
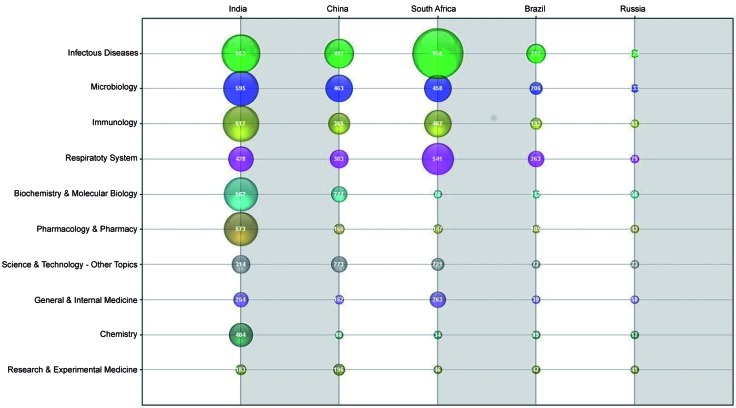
research areas vs BRICS countries (Brazil, Russian, Indian, China, and South Africa) (1993-2016).

In Fig. 8, when analysed proportionally to each country’s production, China shows a steady decline from being the top-ranked article source (infectious diseases, ~17%) to the bottom-ranked article source (chemistry, ~3%). A very similar slope is observed for Brazil and Russia, from the third-ranked downward for each. India and South Africa exhibit two extremes: India reached a plateau at the top of one researched area (infectious diseases, ~14%), but it was fifth in another (biochemistry and molecular biology, ~12%), a decline with a slope similar to those of others; South Africa’s article production was strongly dominated by infectious diseases (~28%) but experienced a fast decline in publications.

## DISCUSSION

Our bibliometric study conducted on BRICS countries from 1993 to 2016 indicated that there has been a rapid increase in publications originating in India since 2000. After 2006, there was a significant rise in the number of publications from China, South Africa, and Brazil. Notably, China’s rate of article production grew after 2010, as described by Nafade et al.^10^ and highlighted by WHO.^6^

Our analysis found that between 1993 to 2016, there was no change in Russia’s apparent rate of TB-related publications. However, this finding cannot be taken at face value. Most scientific articles originating in Russia are not indexed in the Web of Science Core Collection;^19^ even in the days of the Soviet Union, the traditional focus of Russian authors has been to publish in internal Russian journals, which has its own bibliometric and index system, the Russian Science Citation Index (Reviewing the Russian Science Citation Index in WoS, we found a total of 52 articles between 1993-2016).^19^ Nevertheless, the total number of Russian publications indexed by the national bibliometric system for this period was 7,738. Also, there was a visible increase in the number of Russian articles indexed by WoS Core Collection. Over the previous two years, Russian scientific journals, including the main specialised journal *Tuberculosis and Lung Disease*, have been incorporated into Scopus.^21^

It is important to note that citations per country do not necessarily correlate with the volume of publications. For example, many of South Africa’s contributions were collaborations with publications, and these articles were highly cited; this somewhat overstates South Africa’s unique contributions. In contrast, India produced large volumes of publications and received similar numbers of citations, indicating that more seminal research.^10^ ^,^ ^22^

The increase of TB-related articles after 2006 is related to renewed emphasis on TB by WHO,^3^ which gained momentum in the following years with such programs as “The Global Plan to Stop TB 2011-2015: Transforming the Fight Towards Elimination of Tuberculosis” (World Health Organization & Stop TB Partnership ‎2010)‎, although funding gaps led to TB research not being fully incorporated into the Tuberculosis Control Programs in high-burden countries for several years.^23^

Our analysis of the interactions between countries showed that among the BRICS countries, the highest number of interactions occurred between India and South Africa. The interactions were not based solely on language factors; the two countries have the highest TB incidence rate among the BRICS countries.^2^ ^,^ ^4^

According to WHO, in 2016, the incidences were (per 100,000 population): India (211), South Africa (781), Russia (66), China (64), and Brazil (42). When considering BRICS and other countries, there was a clear interaction between South Africa and the United Kingdom and the Netherlands (former colonisers) and the United States, currently the largest funder globally of TB research. However, collaborations in TB research among the BRICS countries occurred less frequently, as noted by Nafadale et al.^10^ and by WHO.^6^ This probably reflects case studies that focus on South Africa.

Our analysis showed that the most productive organisations in TB research were a few public organisations: universities or research institutions. Again, organizations in India and China had the highest productivity, although organisations in South Africa had more interaction with countries outside the BRICS countries (notably, the United Kingdom, the Netherlands, and the United States). In Brazil, the most productive scientific organisations tended to interact with each other; a lesser number interacted with South Africa and India and still fewer interacted with China and Russia. These data underscore the importance of ongoing bibliometric studies to monitor the need for research investment in specific sectors, as well as the need to strengthen areas that still have high levels of TB.^10^ ^,^ ^13^ ^,^ ^15^

As cited by WHO, progress can be attributed in part to increased funding allocations by the United States and the European Union to South Africa and to domestic research funding from sourced within China, India, and South Africa. Given the rising contributions of individual BRICS countries to TB knowledge generation, BRICS countries must allocate more domestic funds to increase collaborative research activities with one another to achieve the goals of the Global End TB Strategy.^5^ ^,^ ^6^

As highlighted by WHO,^6^ 61% of all TB R&D funding from 2009-2015 came from the public sector. Among the top 10 TB research organisations in the BRICS countries, six receive government funds (Fiocruz, UFRJ, and USP in Brazil; AIIMS in India; CDC in China; and the University of Witwatersrand in South Africa), two are private (Stellenbosch University and University of KwaZulu Natal in South Africa), and the University of Cape Town in South Africa is a public-private partnership. Vasconcelos et al.^24^ and Fonseca et al.,^25^ when evaluating TB publications in Brazil, reported that article production has been concentrated in public universities and research institutions. Sweileh et al.,^26^ using the Scopus database, carried out a bibliometric overview of publications on multi-, extensively, and totally drug-resistant TB; they found that three of the high-burden countries for multi-drug-resistant tuberculosis (MDR-TB) - India, China, and South Africa - were also the top article-producing countries.

Our analysis of the research areas and their relationships, despite the limitations inherent to the indicators used in this article, found that publications among the BRICS countries generally covered all research areas, but that infectious diseases, respiratory system, and general and internal medicine were less researched among the publications (43%). However, some countries such as South Africa may not have had the highest number of articles but did have articles of such high quality that the number of citations outweighed the quantity.

Taking into account the research areas covered, there was a low to moderate relationship between BRICS TB publications and the research priorities outlined in the international roadmap for tuberculosis research (the Roadmap) published in 2011 by WHO and the Stop TB Partnership.^27^

Our paper does have some limitations. First, we caution against drawing strong, definitive conclusions from this bibliometric and network analysis because although we analysed the scientific information, we did not analyse the content of every abstract. Second, we should note that analysing only those publications in the English language (as we did) will not have produced a complete or comprehensive picture of the research capacity of each institution among the BRICS countries. Third, searches in other databases such as Scopus, Scival, and Clintrials.gov may have revealed different results; consequently, this analysis may have missed less obvious trends. Fourth, neither the bibliometric nor the network analysis could capture all the needed information, such as citations, to understand current TB research interactions and the collaboration among BRICS researchers. Given the scope of the research areas covered, we could not evaluate the relationships between the BRICS TB articles and the research priorities outlined in WHO’s international roadmap for tuberculosis research and the Stop TB Partnership^27^ because each country had distinct characteristics (e.g., health priorities, budgets, etc.). Fifth, we did not cover publications indexed in social sciences and arts and humanities (Science Citation Index Expanded). Finally, some researchers may have worked with more than one institution; since we only evaluated their first affiliation for our analysis, some information may not have been considered.

The BRICS TB Research Network was launched in 2017,^9^ and further urgent development of a BRICS TB research plan continues, drawing on the resources of India’s TB Research Consortium,^28^ the Brazilian TB Research Network (REDE-TB),^29^ ^)^ the South African Strategic Health Innovation Partnership,^30^ China’s new national spending plan on science and technology,^31^ and the Russian Federation. This approach may increase efforts to ensure that basic science research is successfully translated into products and policies that can help end the TB epidemic. In addition, as highlighted in Moscow at the second BRICS TB Research Network technical meeting in November 2017, increased funding for TB research needs to be supported by strong local scientific leadership, increased research capacity in BRICS countries, thorough transparency, evidence-based approaches, high-quality data, accountability, and knowledge and resource sharing. Only these can strength the BRICS countries’ social, technical, scientific, and industrial capacities to eradicate TB.

## References

[1] United Nations General Assembly (2015). Transforming our world: the 2030 agenda for sustainable
development [cited 2019 September 10]. https://www.unfpa.org/sites/default/files/resourcepdf/Resolution_A_RES_70_1_EN.pdf.

[2] Uplekar M, Weil D, Lonnroth K, Jaramillo E, Lienhardt C, Dias HM (2015). WHO's new end TB strategy. Lancet.

[3] World Health Organization (2015). A global action framework for TB research in support of the third
pillar of WHO's end TB strategy [cited 2019 Septmber 10]. https://www.who.int/tb/publications/global-framework-research/en/.

[4] Pai M (2018). Time for high-burden countries to lead the tuberculosis research
agenda. PLoS Med.

[5] United Nations General Assembly (2018). Political declaration of the high-level meeting of the General
Assembly on the fight against tuberculosis [73/3]. http://www.un.org/en/ga/search/view_doc.asp?symbol=A/RES/73/3.

[6] World Health Organization Global investments in tuberculosis research and development:
past, present, and future. https://apps.who.int/iris/bitstream/handle/10665/259412/9789241513326-eng.pdf?sequence=1.

[7] Kirton J, Larionova M, Alagh Y E (2012). BRICS New Delhi Summit. http://www.newsdeskmedia.com/brics-newdelhi-2012.

[8] Xiaodong W (2017). Tianjin to host 7th BRICS Health Ministers
Meeting. http://www.chinadaily.com.cn/world/2017-06/28/content_29921038.htm..

[9] Raviglione M, Uplekar M, Weil D, Kasaeva T (2018). Tuberculosis makes it onto the international political agenda for
health...finally. Lancet Glob Heal.

[10] Nafade V, Nash M, Huddart S, Pande T, Gebreselassie N, Lienhardt C (2018). A bibliometric analysis of tuberculosis research,
2007-2016. PLoS One.

[11] González-Albo B, Bordons M (2011). Articles vs proceedings papers: do they differ in research
relevance and impact? A case study in the library and information science
field. J Informetr.

[12] Kochi A (1996). Tuberculosis as a global emergency. Kekkaku.

[13] Santoro N, Quattrociocchi W, Flocchini P, Casteigts A, Amblard F (2011). Time-varying graphs and social network analysis: temporal
indicators and metrics. In: 3rd AISB Social Networks and Multiagent Systems
Symposium [Internet]. http://arxiv.org/abs/1102.0629.

[14] Freeman LC (1978). Centrality in social networks conceptual
clarification. Soc Networks.

[15] Wasserman S, Faust K (1994). Social network analysis: methods and applications. Vol.1.

[16] Golbeck J (2013). Analyzing the social web. Waltham: Elsevier Inc.

[17] Blondel VD, Guillaume J, Lefebvre E (2008). Fast unfolding of communities in large networks. J Stat Mech Theory Exp.

[18] Gephi (2010). The Open Graph Viz Platform.

[19] Golbeck J (2013). Analyzing the social web. 1st ed. Waltham: Elsevier Inc.

[20] Jacomy M, Venturini T, Heymann S, Bastian M (2014). ForceAtlas2, a continuous graph layout algorithm for handy
network visualisation designed for the Gephi software. PLoS One.

[21] Gorin SV, Koroleva AM, Ovcharenko NA (2016). The Russian science citation index (RSCI) as a new trend in
scientific editing and publishing in Russia. Eur Sci Ed.

[22] Atkins S, Marsden S, Diwan V, Zwarenstein M (2016). North-south collaboration and capacity development in global
health research in low- and middle-income countries - The ARCADE
projects. Glob Health Action.

[23] Lienhardt C, Espinal M, Pai M, Maher D, Raviglione MC (2011). What research is needed to stop TB Introducing the TB research
movement. PLoS Med.

[24] Vasconcellos AG, Morel CM (2012). Enabling policy planning and innovation management through patent
information and co-authorship network analyses a study of tuberculosis in
Brazil. PLoS One.

[25] Fonseca BPFE, Silva MVPD, Araújo KM, Sampaio RB, Moraes MO (2017). Network analysis for science and technology management evidence
from tuberculosis research in Fiocruz, Brazil. PLoS One.

[26] Sweileh WM, AbuTaha AS, Sawalha AF, Al-Khalil S, Al-Jabi SW, Zyoud SH (2016). Bibliometric analysis of worldwide publications on multi-,
extensively, and totally drug - resistant tuberculosis
(2006-2015). Multidiscip Respir Med.

[27] Brennan Mike, Cobelens F, Lanfranchi B, Lienhardt C, Sizemore C, Waltz G (2011). An international roadmap for tuberculosis research: towards a
world free of tuberculosis. WHO Libr Cat Data.

[28] Government of India (2016). India takes a lead in TB research in a unique mission mode to End
TB.

[29] Kritski A, Barreira D, Junqueira-Kipnis AP, Moraes MO, Campos MM, Degrave WM (2016). Brazilian response to global end TB strategy the national
tuberculosis research agenda. Rev Soc Bras Med Trop.

[30] Samrc (2014). Ship TB projects 2014 SAMRC contact details. http://www.samrc.ac.za/innovation/strategic-health-innovation-partnerships.

[31] Sciencemag (2016). Science is a major plank in China's new spending plan
[Internet]. Science.

