# Supplementary material for: Mapping the tuberculosis scientific landscape among BRICS countries: a bibliometric and network analysis
Source: Mem Inst Oswaldo Cruz. 2020 Mar 16;115:e190342. doi: 10.1590/0074-02760190342 (PMC7066990; doi:10.1590/0074-02760190342)
Supplement: Supplementary file 1 [file 1678-8060-mioc-115-e190342-s.pdf]

TOP 50 organisations - Degree centrality

Organisation	Degree centrality
Univ_Cape_Town_South_Africa	1216
Stellenbosch_Univ_South_Africa	992
Univ_London_Imperial_Coll_Sci_Technol_&_Med_England	811
Johns_Hopkins_Univ_USA	730
Harvard_Univ_USA	696
UCL_England	692
China_CDC_China	685
UNIV_CALIF_SAN_FRANCISCO_SAN_FRANCISCO_CA_USA	642
Fiocruz_Brazil	621
S_African_Med_Res_Council_South_Africa	615
NIH:_NIAID_USA	609
Univ_KwaZulu_Natal_South_Africa	603
AIIMS_India	581
USP_Brazil	522
Fudan_Univ_China	513
WHO_Switzerland	504
Univ_Witwatersrand_South_Africa	501
Univ_Sydney_NSW_Australia	476
Albert_Einstein_Coll_Med_Bronx_NY_USA	461
Karolinska_Inst_Stockholm_Sweden	445
Makerere_Univ_Kampala_Uganda	441
Natl_Inst_Res_TB_India	432
Int_Union_TB_&_Lung_Dis_France	430
Univ_Melbourne_Vic_Australia	420
Univ_Oxford_Oxford_England	417
McGill_Univ_Canada	415
Univ_Washington_Seattle_WA_USA	408
Minist_Hlth_Riyadh_Saudi_Arabia	399
Yale_Univ_New_Haven_CT_USA	397
London_Sch_Hyg_&_Trop_Med_England	388
Univ_N_Carolina_Chapel_Hill_NC_USA	387
Shandong_Univ_China	385
Russian_Acad_Sci_Russia	383
Univ_Groningen_Groningen_Netherlands	382
Univ_Amsterdam_Netherlands	381
Stanford_Univ_Stanford_CA_USA	377
Norwegian_Inst_Publ_Hlth_Oslo_Norway	376
Univ_Bergen_Bergen_Norway	373
Columbia_Univ_New_York_NY_USA	370
EMORY_UNIV_ATLANTA_GA_USA	369
Univ_Massachusetts_Worcester_MA_USA	367
Monash_Univ_Vic_Australia	363
UFRJ_Brazil	361
Natl_TB_Inst_Karnataka_India	361
Post_Grad_Inst_Med_Educ_&_Res_PGIMER_India	358
Chinese_Acad_Med_Sci_China	358
Univ_Minho_Braga_Portugal	358
CDC_USA	356
Christian_Med_Coll_&_Hosp_India	356
Univ_London_London_England	356

Top 30 countries - Degree centrality

Country	Degree centrality
India	130
Canada	129
South_Africa	124
USA	122
Brazil	121
Italy	118
France	116
Australia	116
Philippines	114
Spain	113
Netherlands	113
Indonesia	112
UK	109
China	108
Cameroon	107
ETHIOPIA	106
Portugal	105
Russia	105
Japan	105
Saudi_Arabia	105
Denmark	104
South_Korea	103
UGANDA	99
Argentina	99
Vietnam	98
Mexico	98
Pakistan	95
Tanzania	94
Uruguay	93
Slovakia	92

Top 20 research areas - Degree centrality

ResearchAreas	Degree centrality
Pharmacology_&_Pharmacy	32
Immunology	29
Biochemistry_&_Molecular_Biology	29
Chemistry	26
Pediatrics	24
Pathology	24
Research_&_Experimental_Medicine	21
Biotechnology_&_Applied_Microbiology	21
Surgery	20
Radiology_Nuclear_Medicine_&_Medical_Imaging	20
Computer_Science	20
Biophysics	19
Cell_Biology	19
Infectious_Diseases	18
Engineering	18
Science_&_Technology_-_Other_Topics	17
Oncology	17
Respiratory_System	16
Neurosciences_&_Neurology	16
Microbiology	15
